# Chaotic Signatures of Heart Rate Variability and Its Power Spectrum in Health, Aging and Heart Failure

**DOI:** 10.1371/journal.pone.0004323

**Published:** 2009-02-02

**Authors:** Guo-Qiang Wu, Natalia M. Arzeno, Lin-Lin Shen, Da-Kan Tang, Da-An Zheng, Nai-Qing Zhao, Dwain L. Eckberg, Chi-Sang Poon

**Affiliations:** 1 Department of Mechanics and Engineering Science, Fudan University, Shanghai, People's Republic of China; 2 Harvard-MIT Division of Health Sciences and Technology, Massachusetts Institute of Technology, Cambridge, Massachusetts, United States of America; 3 Department of Electrical Engineering and Computer Science, Massachusetts Institute of Technology, Cambridge, Massachusetts, United States of America; 4 Department of Physiology and Pathophysiology, Fudan University, Shanghai, People's Republic of China; 5 School of Information Science and Engineering, Fudan University, Shanghai, People's Republic of China; 6 School of Public Health, Fudan University, Shanghai, People's Republic of China; 7 Medical College of Virginia at Virginia Commonwealth University, Richmond, Virginia, United States of America; Universidad Europea de Madrid, Spain

## Abstract

A paradox regarding the classic power spectral analysis of heart rate variability (HRV) is whether the characteristic high- (HF) and low-frequency (LF) spectral peaks represent stochastic or chaotic phenomena. Resolution of this fundamental issue is key to unraveling the mechanisms of HRV, which is critical to its proper use as a noninvasive marker for cardiac mortality risk assessment and stratification in congestive heart failure (CHF) and other cardiac dysfunctions. However, conventional techniques of nonlinear time series analysis generally lack sufficient sensitivity, specificity and robustness to discriminate chaos from random noise, much less quantify the chaos level. Here, we apply a ‘litmus test’ for heartbeat chaos based on a novel noise titration assay which affords a robust, specific, time-resolved and quantitative measure of the relative chaos level. Noise titration of running short-segment Holter tachograms from healthy subjects revealed circadian-dependent (or sleep/wake-dependent) heartbeat chaos that was linked to the HF component (respiratory sinus arrhythmia). The relative ‘HF chaos’ levels were similar in young and elderly subjects despite proportional age-related decreases in HF and LF power. In contrast, the near-regular heartbeat in CHF patients was primarily nonchaotic except punctuated by undetected ectopic beats and other abnormal beats, causing transient chaos. Such profound circadian-, age- and CHF-dependent changes in the chaotic and spectral characteristics of HRV were accompanied by little changes in approximate entropy, a measure of signal irregularity. The salient chaotic signatures of HRV in these subject groups reveal distinct autonomic, cardiac, respiratory and circadian/sleep-wake mechanisms that distinguish health and aging from CHF.

## Introduction

Since its introduction in 1981 [1], power spectral analysis of heart rate variability (HRV) has become a standard noninvasive probe of cardiac-autonomic tones [2], [3], [4]. Numerous studies have demonstrated the prognostic power of the high- (HF) and low-frequency (LF) spectral peaks (or their time-domain equivalents [5]) to predict mortality in cardiac patients, especially congestive heart failure (CHF) patients (reviewed in [6], [7]). These spectral components are traditionally characterized using linear Fourier theory and linear models such as transfer function [8], sympathovagal balance ([9], but see [10]) or stochastic point process [11], [12], even though they clearly could also come from nonlinear processes.

In recent years there has been increasing recognition that HRV may in fact represent a much more complex phenomenon reflecting the nonlinear fluctuations of cardiac-autonomic outflows [13], [14], [15] in a fractal [16], [17] or entropic [17], [18], perhaps chaotic manner [19], [20], [21], [22]. The chaotic vs. fractal/entropic/stochastic descriptions of HRV present a dilemma in interpreting its power spectrum. Definitive testing of these divergent characterizations is key to unraveling the physiologic mechanisms underlying HRV, which is critical to its proper use as a noninvasive marker for cardiac mortality risk assessment and stratification in CHF and other cardiac diseases.

However, prevailing tests of chaotic dynamics using myriad nonlinear or complexity measures generally lack sufficient sensitivity, specificity and robustness to discriminate chaos from random noise, much less quantify the chaos level (see Appendix S1 for critique of methods). This is despite the fact that from a practical standpoint, it is not critical whether the detected chaos is completely deterministic or part stochastic so long as it illuminates the underlying deterministic mechanisms [22], [23] (see Appendix S1 for definitions of deterministic chaos and stochastic chaos). Moreover, the limited temporal resolution of many of these methods precludes systematic delineation of any time-dependent variations of the underlying nonlinear or chaotic dynamics of HRV. The limitations of these traditional approaches for nonlinear HRV analysis have led to repeated failures to detect chaos in HRV [24], [25], [26] and lingering controversy as to whether HRV is truly chaotic with strong pathophysiological implications, or sheer stochastic with few mechanistic insights demonstrable beyond the purportedly linear HF and LF peaks [23], [27].

To resolve this fundamental dilemma once and for all, two critical research requirements must be met [23]. First, a quantitative assay with superior sensitivity, specificity and robustness in distinguishing chaos from random noise must be in place. Second, a rich data set must be used that allows for time- and disease-dependent variations of the heartbeat chaos to be discerned and correlated with changes in pathophysiology. Here we employ a unique litmus test for heartbeat chaos based on a novel noise titration assay [28] which has proved to provide a robust, specific and time-resolved measure of the relative chaos level in nonlinear biologic time series [29], [30], [31]. We apply this powerful technique to the analysis of short-segment Holter tachograms from young, elderly and CHF subject groups with known time- and disease-dependent changes in HRV. Our results identified circadian-dependent heartbeat chaos which was linked to the HF component (respiratory sinus arrhythmia, RSA [32]) in young/elderly subjects, and transient heartbeat chaos which was linked to sporadic RR interval spikes. These findings shed new light on the mechanisms of chaotic HRV and their physiologic and pathophysiologic determinants in health, aging and CHF.

## Results

### Circadian rhythms of HRV in health, aging and CHF

**Figure 1:**
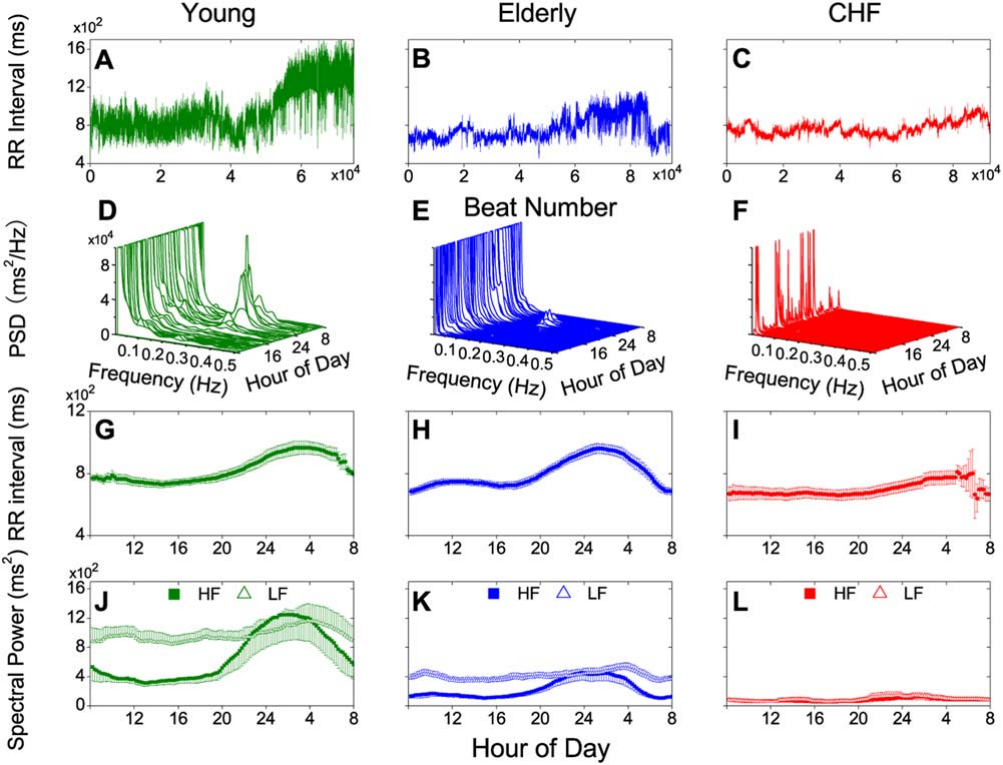
Circadian heart rate variability and mean beat-to-beat (RR) interval in young (green) or elderly subjects (blue) and patients with congestive heart failure (red) as revealed by Holter recordings (sampling rate was 128 Hz for the young group, 250 Hz for the CHF group and 1 kHz for the elderly group). A–C: 24-hr RR interval tachogram in one representative subject from each group. D–F: Corresponding spectral arrays with power spectral density vs. frequency in Hz evaluated by standard autoregression methods [2] at 30-min intervals. G–I: Mean RR intervals (±SE) vs. time for each group. J–L: Corresponding HF (0.15–0.4 Hz) and LF (0.04–0.15 Hz) power. Data points for G–L were evaluated at 12-min intervals and averaged over a moving 3-hr time window for each subject before group averaging.

Figure 1 illustrates the circadian heartbeat rhythms in three subject groups with decreasing HRV: young, elderly and CHF not receiving β-adrenergic blocking drugs. Both the young and elderly groups showed significant nocturnal increases of mean RR interval (Figs. 1A–1B, 1G–1H) and HF power (Figs. 1D–1E and 1J–1K) — changes that mirrored the nocturnal increases of vagal-cardiac tone [33] which mediates the HF component [1], [34], [35], [36], and decreases of sympathetic tone [37] which restrains it [36], [38]. Nocturnal increases of LF power were much lower. The circadian variations of the HF and LF components in healthy and elderly subjects are consistent with those reported previously [39]. These spectral components and their circadian variations were markedly depressed in the parasympathetic-impaired CHF group (Figs. 1F; 1L). The lack of circadian rhythm in the HRV power spectrum of the CHF group was in contrast to the sizable nocturnal increases of the RR interval (Figs. 1C; 1I) possibly reflecting corresponding changes in sympathetic outflow (which is generally augmented in these patients [40], [41]).

### Mean and transient heartbeat chaos

To test whether such circadian-, age- and disease-dependent HRV power spectra signified random noise or chaos, we first discerned nonlinear heartbeat dynamics and discriminated it from background physiologic noise (noise floor) by statistically comparing the goodness of nonlinear vs. linear autoregressive model fits [21], [42] to successive short (12-min) time segments of the 24-hr RR interval series. Once nonlinearity was detected, we then applied a rigorous ‘litmus test’ of chaos by subjecting the data to a novel noise titration assay (see Methods). Because the latter relies on noise titration instead of noise filtering in detecting chaos, it is inherently robust to the attendant noise floor. Indeed, because of inevitable ‘auto-titration’ of experimental data by the background physiologic noise, the detection of nonlinearity alone constitutes a sufficient proof of the presence of chaos [28]. This analytical approach therefore circumvents many of the problems associated with noise contamination of experimental data that frustrate other methods of chaos detection (see Appendix S1).

**Figure 2:**
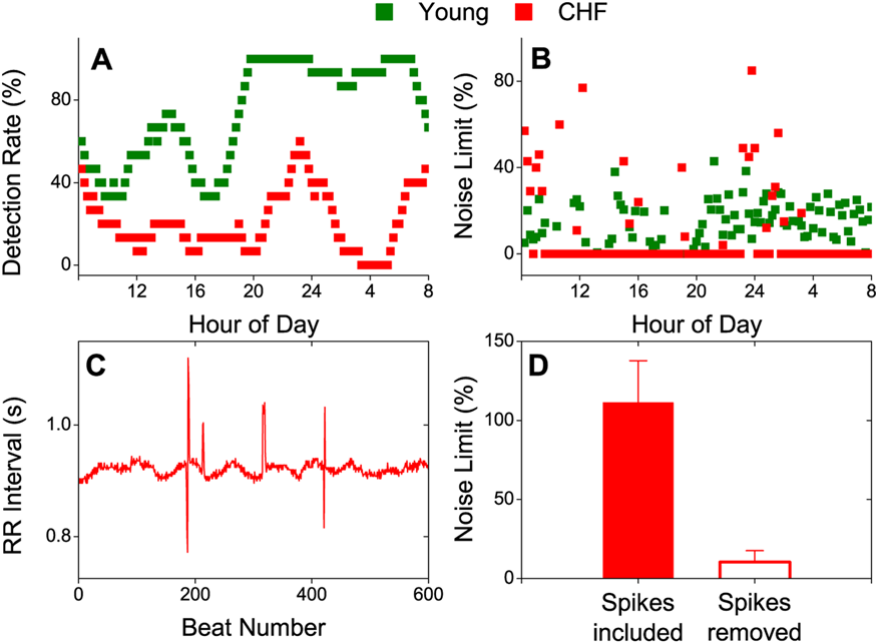
Noise titration assay of mean and transient heartbeat chaos. A. Nonlinear detection rate in a young subject and a CHF patient, evaluated for a moving 3-hr time window with 12-min increments. B. Noise limit in the same subjects evaluated for consecutive 12-min segments without moving-averaging. Time segments where nonlinear dynamics was undetectable are indicated by zero noise limits. C. Example RR interval segment in the CHF patient showing sporadic spikes comprised of undetectable ectopic beats and other abnormal beats such as post-ectopic compensatory pauses (see Methods). The high noise limit of 134.2% in this segment was reduced to 0% (nonlinearity not detected) after the spikes were manually removed. D. Statistical analysis of noise limits with and without (removed) abnormal spikes averaged over 12 nonlinearity-detected segments in 7 CHF subjects showing the highest noise limits.

We used two noise titration measures to assess the changes in chaos level. The detection rate (DR, which gauges the mean chaos level) is defined as the percentage of all time segments in which nonlinearity is detected within a 3-hr time window (see Methods). Figure 2A shows that DR was considerably lower in a CHF patient than a young subject (particularly during nighttime) indicating a decrease of mean heartbeat chaos level in CHF, as reported previously [21].

Next, we estimated the chaos level directly in each segment by using a highly time-resolved measure called the noise limit (NL, see Methods). The latter has been shown [28] to correlate with the equivalent Lyapunov exponent (a gold-standard measure of chaos level [43]) for the noise-free deterministic model except that it measures the relative chaos level (i.e., chaos level less the noise floor) and hence is more robust to noise contamination than numerical estimates of the Lyapunov exponent (see Appendix S1).

Surprisingly, in those segments in which nonlinearity was detected, the measured NLs tended to be higher in the CHF patient than in the young subject (Fig. 2B). The noise titration data in this patient revealed irregular, infrequent, moment-to-moment alternations between abnormally high NLs and zero NLs (nonlinearity not detected) all day long, indicating that HRV vacillated sporadically between chaos and non-chaos (or non-detection of chaos). Close examination of the nonlinearity-detected segments showed that such transient heartbeat chaos was largely the result of RR interval spikes (Fig. 2C, D) comprised of undetected ectopic beats (notwithstanding careful and exhaustive searching efforts; see Methods) and other abnormal beats such as post-ectopic pauses.

### Chaotic vs. spectral and entropic measures of HRV: circadian and CHF-dependent effects

**Figure 3:**
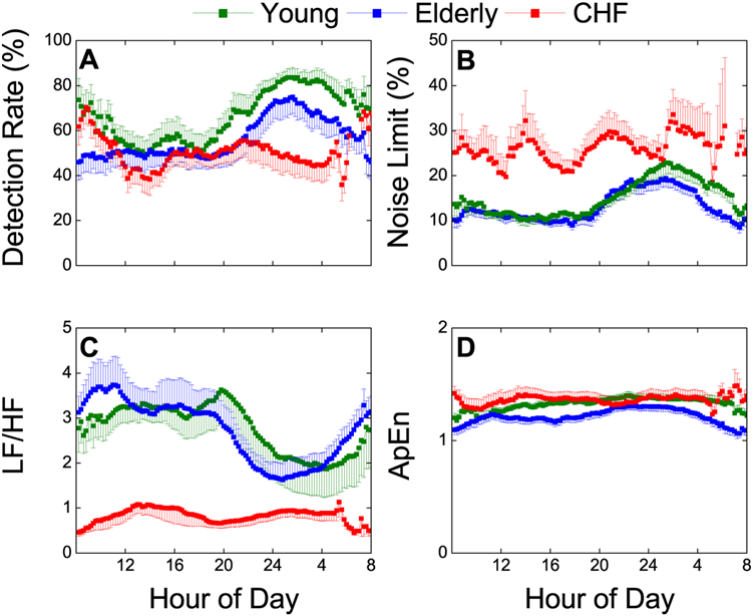
Chaotic vs. spectral and stochastic measures of heart rate variability in young, elderly and CHF groups. A. Detection rate (DR). B. Noise limit (NL). C. LF/HF power ratio. D. Approximate entropy (ApEn). The average DR and NL and LF/HF values were not significantly different between the young and elderly groups (repeated measures ANOVA, P>0.1) but were markedly different in the CHF group. The elderly group had significantly different ApEn values than the young group (P = 0.02) and CHF group (P = 0.04) but the difference between the young and CHF groups were marginal (P = 0.61).

To compare these chaotic measures with traditional measures of HRV, we calculated the corresponding LF to HF power ratio (LF/HF, a measure of relative HRV spectral power [2], [9]) and approximate entropy (ApEn, a measure of signal irregularity [44], [45]) in the three subject groups. As shown in Figs. 3A–3B, DR and NL were not significantly different in the young and elderly groups, and both exhibited similar diurnal/nocturnal variations, suggesting that the relative chaos level of the normal heartbeat was circadian-dependent and not influenced by aging. These salient characteristics of DR and NL were mirrored by LF/HF in a reciprocal manner, again with no significant differences between the young and elderly groups (Fig. 3C).

In comparison, the CHF group demonstrated significantly lower DRs yet higher NLs throughout much of the day (Figs. 3A–3B), indicating decreased mean and increased transient heartbeat chaos levels with little circadian variations (cf. Fig. 2). LF/HF was also significantly lower and with little circadian variations in this group (Fig. 3C).

By contrast, ApEn did not vary significantly throughout the day in all groups and was marginally different between the young and CHF groups (Fig. 3D) despite profound circadian-, age- and CHF-dependent changes in HRV chaotic and spectral characteristics (Figs. 3A–3C). Thus, ApEn is not a sensitive or specific measure of heartbeat chaos or a marker of its physiologic and pathophysiologic determinants.

### HF chaos in health and aging vs. transient heartbeat chaos in CHF

**Figure 4:**
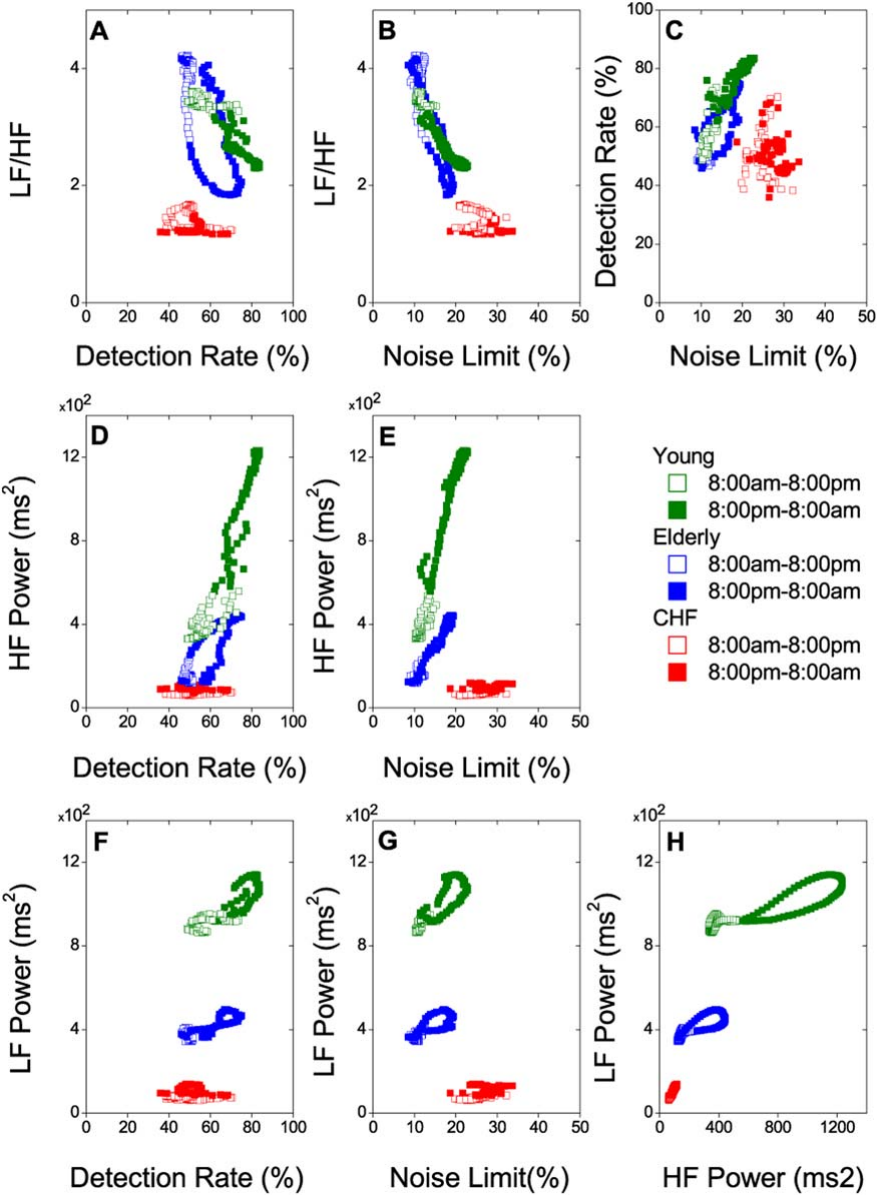
Correlations between chaotic and spectral indices of HRV. Data are average values from each group. Open symbols, daytime; filled symbols, nighttime.

Next, we examined the relations between these chaotic and spectral measures of HRV in the three subject groups. LF/HF correlated negatively with DR (Fig. 4A) and NL (Fig. 4B) in the young/elderly groups (and only weakly so in the CHF group). DR correlated strongly and positively with NL in the young/elderly groups but not the CHF group (Fig. 4C), further demonstrating its significance as a measure of mean but not transient heartbeat chaos level. Both DR and NL correlated only weakly (or not at all) with HF and LF in the CHF group (Figs. 4D–4G), in agreement with the transient nature of the heartbeat chaos in CHF.

By contrast, DR and NL correlated strongly and positively with HF power and only weakly with LF power in the young/elderly groups (Figs. 4D–4G), hence explaining their corresponding negative correlations with LF/HF (Figs. 4A–4B). These data when taken together show that RSA accounted for much of the heartbeat chaos in the young and elderly subjects. Although the LF component might also contribute a minor fraction of the chaotic dynamics as indicated by its (relatively weak) correlations with DR and NL, such correlations could be secondary to its circadian parasympathetic-mediated covariation with the HF component instead (Fig. 4H). Hence, we refer to the chaotic dynamics of RSA in young/elderly subjects as ‘HF chaos.’

### Circadian chaotic and spectral discriminants of HRV in health, aging and CHF

All subject groups could be readily discriminated by the circadian variations of the chaotic and spectral characteristics of HRV (Figs. 3–4). Either DR or NL or LF/HF alone effectively distinguishes the young/elderly groups from the CHF group during specific times of the day: nighttime for DR and daytime for NL and LF/HF (Figs. 3A–3C; 4A–4C). All three groups could be distinguished from one another based on HF or LF power alone throughout much of the day (Figs. 1J–1L) but more effectively so when combined with DR or NL (Figs. 4D–4G), or when HF and LF were plotted together (Fig. 4H). The separation of groups was most clear-cut when LF was plotted against DR, NL or HF during either daytime or nighttime or both (Figs. 4F–H). Groups can be teased apart even further when the circadian variations of multiple chaotic and spectral measures are plotted against one another in higher dimensions (not shown). Note that the discrimination of the subject groups demonstrated here takes into account the temporal (circadian or transient) variations of the chaotic and spectral characteristics of HRV and, hence, is inherently more sensitive than previous fractal-based discrimination approaches that relied on 24-hr HRV data [18], [46].

## Discussion

The present study distinguishes itself from all previous studies of HRV by employing a litmus test for heartbeat chaos and applying it to three subject groups with characteristic circadian- and disease-dependent changes in the HRV power spectrum that are closely related to the underlying pathophysiology. In contrast to traditional nonlinear or complexity methods that lack sufficient sensitivity, specificity and robustness in discriminating chaos from random noise (see Appendix S1), the noise titration approach allowed us to not only discern heartbeat chaos but characterize it in a quantitative manner with great temporal and magnitude resolutions, making it possible for the first time to correlate the circadian- and disease-dependent changes in heartbeat chaos with corresponding changes in HRV and its power spectrum. As discussed below, our results reveal distinct chaotic signatures of HRV which can be ascribed to specific physiologic and pathophysiologic mechanisms that distinguish health and aging from CHF.

### Chaotic signatures of HRV

**Figure 5:**
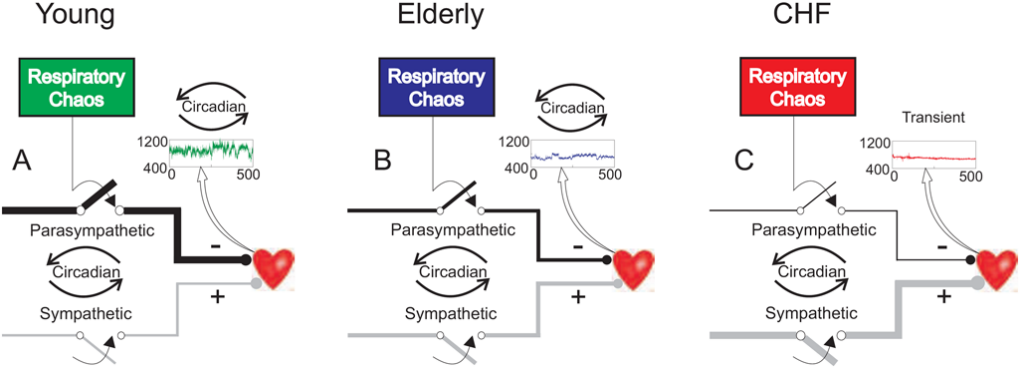
Mechanisms of high-frequency (HF) and transient heartbeat chaos in young, elderly and CHF subjects. Insets show representative 500-beat segments of RR interval series. A. In young subjects, chaotic respiratory rhythmic activity [29] introduces HF chaos in the heartbeat via preganglionic temporal gating (denoted by switches) of vagosympathetic outflows [35] and other mechanisms, resulting in respiratory sinus arrhythmia. Respiration confers the chaotic dynamics of the HF component whereas vagal-cardiac tone (indicated by line thickness) sets the chaos level (HF power) relative to noise floor (LF power). Hence LF/HF is reciprocal to the S/N of HF chaos. Parasympathetic outflow slows (−) the heart rate but increases HF power, whereas sympathetic outflow does the opposite (+). Nocturnal increases of parasympathetic outflow and decreases of sympathetic outflow result in corresponding circadian (curved arrows) variations of the S/N of HF chaos and hence, DR and NL. B. Elderly subjects exhibit proportionate decreases in parasympathetic-mediated HF and LF power, with almost invariant LF/HF compared with young subjects (Figs. 1K, 2E). DR and NL are similar to those of young subjects because the effective S/N are largely unchanged even though the intensity of HF chaos (in terms of the HF power) decreases with age. C. In CHF patients, parasympathetic-mediated circadian HF and LF power are greatly suppressed and transient heartbeat chaos intrinsic to cardiac dynamics emerges.

Our noise titration results provide the strongest evidence yet that HRV in health and in CHF indeed demonstrates chaotic dynamics — but one is circadian- (or sleep/wake)-dependent and the other transient. In both circumstances the HRV proves chaotic even though it is currently not possible to reliably differentiate whether the chaos was completely “deterministic” or part “stochastic” (see Appendix S1). Such semantic issues aside, it is clear that the circadian-, age- and CHF-dependent chaotic signatures of HRV presently identified are not mere stochastic phenomena and must involve a strong deterministic component, as they are seen to correlate closely with time-, aging- and disease-dependent changes in HRV and its power spectrum that were not tracked by ApEn. These observations shed new light on the physiologic and pathophysiological determinants of heartbeat chaos that distinguish health and aging from CHF (Fig. 5).

### Mechanism of HF chaos in health and aging

#### LF/HF as inverse signal-to-noise ratio (S/N) of HF chaos

Our results suggest that the chaos in the normal heartbeat is ascribable largely to RSA. This finding is consistent with preliminary data which showed that the HF component in healthy subjects remained chaotic (with a significant NL) even when all other components were band-phase-randomized, but not vice versa [47]. For short data segments (12 min duration), the very- or ultra-low-frequency components have only minimal effect on HRV. The LF component therefore constitutes a physiologic noise floor that auto-titrates the HF chaos when evaluating short-segment DR and NL in healthy subjects. Thus LF/HF, as a normalized measure of HRV power spectrum, also provides a nonlinear measure that is roughly reciprocal to the S/N of HF chaos in this case — as indicated by the strong negative correlations of LF/HF with both DR and NL in the young/elderly groups (Figs. 3A–3C, 4A–4B). It should be noted, however, that significant chaotic dynamics may potentially also exist in the LF component in other circumstances. Thus LF/HF is not a universal measure of S/N of HF chaos and its significance must be carefully scrutinized from case to case.

#### Respiratory and vagal-cardiac determinants of HF chaos level

Such HF chaos in young/elderly subjects is ascribable at least in part to the recently reported chaotic dynamics of respiratory activity (again discerned by noise titration) [29], [31] — which may induce RSA via its gating of preganglionic vagal-cardiac neural activity [35] (and perhaps to some extent also via modulation of the baroreflex [48]). A significant contribution of respiratory chaotic dynamics to heartbeat chaos is also supported by the recent finding that the NL of HRV in healthy young subjects is strongly modulated by voluntary breathing, such as during speech or breath holding [48]. Recent modeling studies have confirmed that the pacemakers responsible for generating the respiratory rhythm [49] are indeed capable of producing a chaotic burst pattern characterized by a positive NL and sensitive dependence on initial conditions [30]. The available evidence and present results, when taken together, suggest that respiratory activity most likely confers the chaotic dynamics of RSA whereas cardiac-autonomic tones modulate the resultant chaos level (Fig. 5A). According to this model, nocturnal increases of HF power (Figs. 1J–1K) mediated by increases in vagal inhibition and decreases in sympathetic opposition [36], [38] result in increases in S/N of HF chaos (as indicated by corresponding decreases in LF/HF; Fig. 3C) and hence, in DR and NL (Figs. 3A–3B). This model therefore explains (for the young/elderly groups) the observed strong circadian correlations of DR and NL with the HF component, and their negative circadian correlations with LF/HF.

The circadian parasympathetic-sympathetic modulation of HF chaos may be intrinsic to the circadian clock or secondary to the sleep-wake cycle – including changes in sleep stages, changes from supine to upright postures during the sleep-wake cycle, or changes in diurnal/nocturnal respiratory patterns. The relative contributions of these factors to the diurnal-nocturnal changes in HF chaos should be addressed in future studies.

#### Age-invariance of relative HF chaos

This model also explains why DR and NL were relatively unaffected by aging despite corresponding decreases in HRV (Fig. 5B). The observed proportionate decreases of HF and LF power with aging (Figs. 1J–1K) in the face of normal circadian variations of RR interval (Figs. 1G–1H) likely reflect aging-related blunting of baroreflex sensitivity [40], [50], which may influence the LF component [51] as much as the HF component (also see Figs. 1J–1K) [52]. Consequently, despite significant age-dependent decreases in HF chaos level (as measured by the HF power), the S/N of HF chaos (as measured by the reciprocal of LF/HF) remains largely unchanged. Such aging-related parallel decreases of the HF and LF components keep the relative HF chaos level (as measured by DR and NL) relatively age-invariant.

### Mechanism of transient heartbeat chaos in CHF

#### Absence of HF chaos in CHF

The present results confirm that DR is decreased in CHF compared with healthy subjects, as reported previously [21]. The difference in DR was most prominent during nighttime when parasympathetic/sympathetic-dependent HF chaos in the young/elderly subjects climaxed. Importantly, the present results show that the decreased DR in CHF reflects an absence of HF chaos, an effect which is unrelated to aging but represents a direct consequence of impaired vagal-cardiac mediation of RSA.

#### Transient heartbeat chaos in CHF

In addition, our noise titration results reveal pronounced transient heartbeat chaos precipitated by sporadic RR interval spikes, which are intrinsic to the abnormal cardiac dynamics in CHF (Fig. 5C). Such transient chaos was evident even after our careful and exhaustive removal of the great bulk of premature beats (see Methods). Presumably, such transient chaos would be even more pronounced and perhaps more complex (such as high-dimensional chaos), had all premature beats been included in our assay.

Traditionally, HRV has been used primarily as a probe of autonomic regulation; any attendant ectopic beats are generally considered as artifacts to be rid of, although their elimination often proves challenging especially in bulk [53]. In CHF, HRV is greatly suppressed and is marred by excessive and often undetected ectopic beats, and hence is not readily amenable to quantitative analysis. Previous studies using Poincaré plots or other graphical approaches demonstrated erratic HRV with increased complexity in some elderly subjects [54] and CHF patients [55], particularly those with marked sympathetic activity [55], [56]. However, it is not clear whether such erratic HRV pattern was due to undetected ectopic beats and whether it represented increased HF chaos, transient chaos, or increased random noise instead. The present results suggest that in CHF, transient chaos of HRV resulting from sporadic undetected ectopic beats or other abnormal beats (such as post-ectopic compensatory pauses) *per se* may be of greater import than normal HF chaos (which is greatly attenuated in CHF), in that the temporal evolution of such transient chaos may reveal valuable information about abnormal cardiac function that cannot be gleaned from examination of the ECG or changes in the RSA or HF chaos alone (Fig. 5C). The contributions of varying types of ectopic beats and other abnormal beats (such as post-ectopic compensatory pauses) to heartbeat chaos and other erratic HRV patterns in CHF and their underlying pathophysiologic mechanisms deserve further study.

### Conclusion

In conclusion, noise titration provides a robust, specific, time-resolved and quantitative assay for heartbeat chaos that is superior to ApEn and other nonlinear, fractal or entropic methods of HRV analysis. Our results based on this powerful analytical approach show that: (1) HRV in healthy young or elderly subjects indeed exhibits circadian- (or sleep-wake)-dependent chaos that is linked to RSA or the HF component. (2) The HF chaos level (relative to background physiologic noise) discerned by noise titration is modulated by changes in cardiac autonomic tones and is inversely proportional to LF/HF in healthy subjects regardless of age. (3) In CHF patients, HF chaos is absent but transient chaos emerges due to undetected ectopic beats and other abnormal beats such as post-ectopic compensatory pauses, which provides a new measure of abnormal cardiac function in CHF. (4) The salient chaotic signatures of HRV in these subject groups reveal distinct autonomic, cardiac, respiratory and circadian/sleep-wake mechanisms that distinguish health and aging from CHF. These findings provide a mechanistic and quantitative basis for the proper use of the chaotic and spectral characteristics of HRV as noninvasive markers for cardiac mortality risk assessment and stratification in CHF and other cardiac dysfunctions in future.

## Methods

### Ethics statement

The study had been prior reviewed and approved by the MIT Committee on the Use of Humans as Experimental Subjects. All human subject data were analyzed and reported anonymously.

### Subjects

Recordings for the young group (n = 13, age 32±8 yrs mean±SD) and CHF group (n = 14, 56±12 yrs, all New York Functional Classes III and IV and treated with conventional medical therapy but not β-adrenergic blockers) were from the PhysioNet database [57]. Recordings for the elderly group (n = 16, 56±10 yrs, some with suspected coronary artery disease but all with no history of myocardial infarction, CHF, respiratory dysfunction or diabetes) were from one of our own laboratories (see Appendix S2 for further details and Appendix S3 for a listing of individual 24-hr RR interval data). Young subjects were significantly younger (*P*<0.001, LSD *t* test) than elderly subjects and CHF patients but the ages of the latter groups were not significantly different (*P*>0.1, LSD *t* test).

### Extraction of RR intervals

Subjects in all groups were selected on the basis of stability of the mean heart rate and limited number of ectopic beats and undetected beats. Heartbeat intervals were extracted from annotated Holter recordings by using an algorithm (Cygwin) provided by PhysioNet which allowed elimination (without interpolation) of premature or missing beats and other ectopic beats [57]. To minimize recognition artifact [58], all remaining ectopic beats were further examined and any residual premature beats with abnormal QRS complexes undetected by preprocessing were manually removed. For CHF subjects, this combination of automated and manual preprocessing eliminated most of the premature beats and the resultant RR intervals were highly regular with only sporadic ectopic beats (undetected premature beats) and other abnormal beats such as post-ectopic compensatory pauses.

### Heart rate variability analyses

#### Spectral analysis

The power spectral density of the RR interval series was computed (without resampling) by using a linear autoregressive model of order 15 (Burg algorithm) as recommended by the Task Force [2]. The frequency unit for each series was converted from cycles/beat to Hz by division with the average RR interval [9], i.e. Hz = (c/b)/(mean RRI). Spectral components were evaluated as the integral of power spectral density within the LF band (0.04–0.15 Hz) and HF band (0.15–0.4 Hz).

#### Chaotic analysis

The method of noise titration [28] offers a highly sensitive litmus test (sufficient proof) for chaotic dynamics and a relative measure of the chaos level in short, noise-contaminated data segments. In this method, nonlinear determinism in a time series was first identified [21], [42] by comparing a linear model and a polynomial autoregressive model (Eq. 1) with varying memory order (*κ*) and nonlinear degree (*d*) to optimally predict the data based on the Akaike information criterion *C(r)* (Eq. 2):
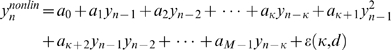
(1)

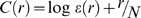
(2)


In the above, *ε* is the modeling error; *M* is the total number of polynomial terms in Eq. 1; *r* is the number of leading polynomial terms in Eq. 1 (1<*r*<*M*) used in the computation of *C(r)*; *N* is the total number of data points in the series. The parameters *a_m_* in Eq. 1 were recursively estimated. The null hypothesis — a stochastic time series with linear dynamics — was rejected if the best nonlinear model provided a significantly better fit to the data than the best linear model using parametric (F-test) statistics at the 1% significance level.

Once nonlinear determinism was indicated, white noise of increasing standard deviations was added to the data until nonlinearity could no longer be detected, i.e., the nonlinearity was ‘neutralized’. The noise limit (NL) was calculated as the percent of signal power added as noise to ‘titrate’ the data to the point of neutrality. Typically, an average NL value was obtained by repeating the titration procedure 5–10 times. Under this scheme, chaos is indicated by NL>0, and the value of NL provides a relative measure of chaos intensity. Conversely, if NL = 0, then it may be inferred that the series either is not chaotic or the chaotic component is already neutralized by the background noise (noise floor) in the data [28].

#### Complexity analysis

Approximate entropy (ApEn) was calculated as described elsewhere [45], [59] as a measure of the irregularity of a time series. We used a standard ApEn(2, *δ*, *N*) index with an embedded dimension of 2 and a tolerance of *δ* (usually 0.1–0.25, here 0.2), which are typical for HRV analysis.

### Data segmentation

To account for the nonstationarity of HRV, 24-hr heartbeat data were divided into 120 segments (12 min each) with ∼800 beats per segment, as with previous studies [21]. Nonlinear detection rate (DR) was calculated as the percentage of detected nonlinear segments in a 3-hour window (with 15 segments for percentile calculation) during the specific period. The window was centered on the present segment and moved one segment at a time. The values of LF, HF, NL and ApEn were calculated segment-by-segment and averaged over 15 segments in a similar fashion (the calculation of average NL excluded all linear segments, such that segments with NL = 0 were not included in the average).

## Supporting Information

Appendix S1 Critique of chaos detection methods
(0.12 MB DOC)

Appendix S2 Description of the elderly group
(0.05 MB DOC)

Appendix S3 Individual elderly subjects data (Zip file)
(2.51 MB ZIP)
