# Supplementary material for: Chaotic Signatures of Heart Rate Variability and Its Power Spectrum in Health, Aging and Heart Failure
Source: PLoS One. 2009 Feb 2;4(2):e4323. doi: 10.1371/journal.pone.0004323 (PMC2629562; doi:10.1371/journal.pone.0004323)
Supplement: Appendix S1 — Critique of chaos detection methods (0.12 MB DOC) [file pone.0004323.s001.doc]

**Appendix S1: Critique of chaos detection methods**

**Detecting deterministic chaos in the presence of measurement noise**

A presumed gold standard test for chaos in a “deterministic” time series is the positivity of its largest Lyapunov exponent indicating sensitive dependence on initial conditions, which is a hallmark of chaos [1]. Although the largest Lyapunov exponent can be readily estimated using established algorithms (e.g. [2,3]), this approach is generally not reliable when the data series is relatively short and/or corrupted by sizable measurement noise, which is often the case for empirical data. A more robust and sensitive approach is to test for nonlinear determinism of the series by using the surrogate data method [4,5] or nonlinear autoregressive modeling method [6]. However, a major drawback of this approach is that nonlinear determinism is only a necessary and qualitative criterion for deterministic chaos, and neither of these methods *per se* allows a sufficient proof of chaos or provides a quantitative measure of chaos intensity.

This fundamental difficulty in detecting deterministic chaos is rectified by the noise titration method, which takes advantage of the specificity, sensitivity and robustness properties of the nonlinear autoregressive modeling method to provide a quantitative estimate of the relative Lyapunov exponent in short, noisy data [7]. By adding artificially generated random noise to quantitatively titrate chaos under the nonlinear determinism test, this procedure is inherently robust to measurement noise. Indeed, the inevitable presence of measurement noise allows auto-titration of the data by the noise floor. The noise titration method therefore provides a simple litmus test for sensitive, specific, and robust detection of deterministic chaos as well as quantitative measurement of chaos intensity in short, noisy data.

**Deterministic chaos vs. stochastic chaos or complexity**

In addition to spontaneous (autonomous) deterministic chaos, a nonlinear dynamic system may also be driven into (or out of) the chaotic regime by appropriately chosen deterministic or stochastic inputs (see discussion in [7]). Such induced chaos may still be considered “deterministic chaos” so long as the input is deterministic, whether the input is itself chaotic or not. For stochastic inputs (“dynamic noise”) the resultant noise-induced chaos represents “stochastic chaos” (reviewed in [8]). A generalized Lyapunov exponent for assessing both deterministic and noise-induced chaos has been proposed [9], and both noise-induced chaos [10] and noise annihilation of deterministic chaos [6] can be detected by the nonlinear autoregressive model for noise titration, as suggested previously [7]. There is currently no available technique to readily distinguish spontaneous or induced deterministic chaos from noise-induced chaos or noise annihilation of deterministic chaos in nonautonomous nonlinear dynamic systems. Nevertheless, inasmuch as the chaotic dynamics of a time series can be reliably quantified by noise titration or other means and shown to correlate with meaningful system mechanisms (in this case NL or DR vs. the HF component of HRV or sporadic RR interval spikes), the precise mathematical classification of the chaos (spontaneous or induced, deterministic or stochastic) or nonchaos is unimportant (see discussions in [11,12]). Indeed, the present results suggest that the circadian heartbeat chaos in healthy subjects reflects predominantly RSA induced by chaotic respiratory rhythm, whereas the transient heartbeat chaos in CHF is probably spontaneous and intrinsic to abnormal cardiac dynamics rather than induced by respiratory or vagal-cardiac inputs.

On the other hand, various fractal or entropic measures have been used to analyze the “complexity” of HRV without regard to the underlying deterministic or stochastic processes, whether chaotic or not. These complexity measures generally lack the ability to reveal salient nonlinear dynamics and their relationships with the underlying physical or biological mechanisms, even for a known deterministic model. For example, monofractal measures such as 1/*f* scaling or long-range detrended fluctuation analysis (DFA) were found to be poor indicators of nonlinear determinism in HRV based on standard amplitude-adjusted phase randomization surrogate data method [13], even though they were adequate for shuffled surrogates [13,14]. Since shuffled surrogates wipe out all linear and nonlinear correlations whereas amplitude-adjusted phase-randomized surrogates judiciously retain the linear correlations of the original time series [4,5], it follows that 1/*f* scaling and DFA are essentially linear predictor statistics and cannot distinguish linear from nonlinear correlations. This observation should not be surprising considering that 1/*f* scaling pertains specifically to the Fourier amplitude spectrum, whereas information about nonlinear correlations is contained exclusively in the phase spectrum [5]. Similar argument also applies to DFA, which pertains to fluctuations in magnitude but not in phase relations of the times series. Since DFA represents the time-domain equivalent of 1/*f* scaling [15], it too cannot distinguish linear and nonlinear correlations. Similarly, classical entropic measures such as ApEn has been shown to be nonrobust to measurement noise in detecting deterministic chaos [13]. In the present study, ApEn proved to be inferior to NL and DR in characterizing the HRV power spectrum. Similar limitations also apply to sample entropy (a refined variant of ApEn [16]) or other complexity measures such as Shannon entropy [17,18], a mono-entropic measure that is related to ApEn [19,20].

Recently, higher-order complexity measures based on multifractal [14] or multiscale entropy [21] methods have been proposed to purportedly remedy the shortcomings of monofractal and mono-entropic measures, although they do not necessarily perform any better either [17,22]. These elaborate approaches generally require minimal measurement noise with much longer data lengths and are computationally intensive with limited temporal resolution, and are fraught with many pitfalls [17,23]. Furthermore, these graphical methods provide only qualitative (instead of quantitative) and non-statistical assessment of HRV and, as with other complexity measures, do not discriminate (deterministic or stochastic) chaos from nonchaotic nonlinear dynamics or measurement noise, unlike NL and DR.

In summary, although many nonlinear or complexity methods have been shown to distinguish healthy heartbeat dynamics from those with CHF even if age is taken into account, they all lack the specificity, sensitivity, temporal resolution, robustness and quantitative power necessary for tracking the time-dependent changes in HRV and in its power spectrum to illuminate the underlying physiologic and pathophysioloic mechanisms as reported here.

**REFERENCES**

1. Eckmann J-P, Ruelle D (1985) Ergodic theory of chaos and strange attractors. Reviews of Modern Physics 57: 617-656.

2. Briggs K (1990) An Improved Method for Estimating Liapunov Exponents of Chaotic Time-Series. Physics Letters A 151: 27-32.

3. Rosenstein MT, Collins JJ, Deluca CJ (1993) A practical method for calculating largest Lyapunov exponents from small data sets. Physica D 65: 117-134.

4. Schreiber T, Schmitz A (2000) Surrogate time series. Physica D-Nonlinear Phenomena 142: 346-382.

5. Theiler J, Eubank S, Longtin A, Galdrikian B, Farmer JD (1992) Testing for Nonlinearity in Time-Series - the Method of Surrogate Data. Physica D 58: 77-94.

6. Barahona M, Poon CS (1996) Detection of nonlinear dynamics in short, noisy time series. Nature 381: 215-217.

7. Poon CS, Barahona M (2001) Titration of chaos with added noise. Proc Natl Acad Sci U S A 98: 7107-7112.

8. Deng Z-D, Massachusetts Institute of Technology. Dept. of Electrical Engineering and Computer Science. (2007) Stochastic chaos and thermodynamic phase transitions : theory and Bayesian estimation algorithms [Thesis M. Eng. and S.B. --Massachusetts Institute of Technology Dept. of Electrical Engineering and Computer Science 2007.]. 200 p. p.

9. Gao JB, Hu J, Tung WW, Cao YH (2006) Distinguishing chaos from noise by scale-dependent Lyapunov exponent. Physical Review E 74: 066204.

10. Lei M, Meng G (2008) The influence of noise on nonlinear time series detection based on Volterra-Wiener-Korenberg model. Chaos Solitons & Fractals 36: 512-516.

11. Poon CS (1999) Cardiac chaos: implications for congestive heart failure. Congest Heart Fail 5: 270-274.

12. Poon CS (2000) The chaos about heart rate chaos. J Cardiovasc Electrophysiol 11: 235-236.

13. Li C, Tang D-K, Zheng D-A, Ding G-H, Poon C-S, et al. (2008) Comparison of nonlinear indices in analyses of heart rate variability. Conf Proc IEEE Eng Med Biol Soc. Vancouver, British Columbia, Canada. pp. 2145-2148.

14. Goldberger AL, Amaral LA, Hausdorff JM, Ivanov P, Peng CK, et al. (2002) Fractal dynamics in physiology: alterations with disease and aging. Proc Natl Acad Sci U S A 99 Suppl 1: 2466-2472.

15. Beran J (1994) Statistics for long-memory processes. New York: Chapman & Hall. x, 315 p. p.

16. Richman JS, Moorman JR (2000) Physiological time-series analysis using approximate entropy and sample entropy. Am J Physiol Heart Circ Physiol 278: H2039-2049.

17. Wessel N, Malberg H, Bauernschmitt R, Kurths J (2007) Nonlinear methods of cardiovascular physics and their clinical applicability. International Journal of Bifurcation and Chaos 17: 3325-3371.

18. Letellier C (2006) Estimating the Shannon entropy: Recurrence plots versus symbolic dynamics. Physical Review Letters 96: 254102.

19. Pincus SM (1991) Approximate entropy as a measure of system complexity. Proc Natl Acad Sci U S A 88: 2297-2301.

20. Pincus SM, Goldberger AL (1994) Physiological time-series analysis: what does regularity quantify? Am J Physiol Heart Circ Physiol 266: H1643-1656.

21. Costa M, Goldberger AL, Peng CK (2005) Multiscale entropy analysis of biological signals. Phys Rev E Stat Nonlin Soft Matter Phys 71: 021906.

22. Nikulin VV, Brismar T (2004) Comment on "Multiscale entropy analysis of complex physiologic time series". Physical Review Letters 92: 089803.

23. Veneziano D, Moglen GE, Bras RL (1995) Multifractal analysis: Pitfalls of standard procedures and alternatives. Physical Review E Statistical Physics, Plasmas, Fluids, and Related Interdisciplinary Topics 52: 1387-1398.
