# Supplementary material for: Chaotic Signatures of Heart Rate Variability and Its Power Spectrum in Health, Aging and Heart Failure
Source: PLoS One. 2009 Feb 2;4(2):e4323. doi: 10.1371/journal.pone.0004323 (PMC2629562; doi:10.1371/journal.pone.0004323)
Supplement: Appendix S2 — Description of the elderly group (0.05 MB DOC) [file pone.0004323.s002.doc]

**Appendix S2: Description of the elderly group**

**Elderly group**

| No | sex | age | start | Symptom | Date |
| --- | --- | --- | --- | --- | --- |
| Z931913 | M | 63 | 10:10 |  |  |
| Z932186 | M | 62 | 9:00 |  |  |
| Z941806 | M | 45 | 8:00 |  |  |
| Z941816 | M | 50 | 8:10 |  |  |
| Z942166 | M | 65 | 8:50 |  |  |
| Z95321 | M | 65 | 8:15 |  |  |
| Z96926 | M | 50 | 8:35 |  |  |
| Z96954 | M | 45 | 9:15 |  |  |
| ZD93824 | M | 68 | 8:35 |  |  |
| ZD951395 | M | 57 | 14:15 |  |  |
| ZD951574 | F | 72 | 8:30 |  |  |
| ZH11292 | F | 51 | 8:55 | angina | 06/27/1994 |
| ZH11361 | F | 60 | 9:00 | angina | 07/02/1994 |
| ZH11395 | F | 41 | 8:15 | angina | 07/06/1994 |
| ZH11417 | F | 42 | 8:52 | angina | 07/04/1994 |
| ZH11424 | M | 58 | 8:30 | angina | 07/08/1994 |

The inclusion criteria for elderly subjects were:

1. No history of myocardial infarction, cancer, or diabetes;
2. No chronic respiratory disease;
3. Fewer than 100 identifiable premature beats in a 24-hour electrocardiogram (ECG) recording;
4. Normal chest X-ray;
5. Normal results from biochemical examination of urine and blood;
6. Age between 45 and 80 years;
7. Non-smoking;
8. Some subjects had evidence suggestive of myocardial ischemia during 24-hour electrocardiogram recordings: horizontal or downsloping ST-segments greater than 1 mm 60 ms after the J point, during more than 7 consecutive complexes.

The exclusion criteria were:

1. Any acute respiratory illness (including the common cold);
2. Any pain or obvious skin disease;
3. Abnormal body temperature;
4. Left ventricular hypertrophy or conduction abnormalities;
5. Poor sleep quality, or less than 7 hours sleep;
6. Congestive heart failure;
7. Systolic pressure > 145 mm Hg or < 110 mmHg;
8. Diastolic pressure > 90 mmHg or < 55 mmHg;
9. Beta-adrenergic blocking therapy within the past two weeks.

**ECG data collection and processing for the elderly group**

The 24-hour ECG tape recording for each elderly subject was recorded from 8:30 AM to 8:30 AM the next day in Zhongshan Hospital, Shanghai, China. Each 24-hour ECG signal was replayed by a recorder with two output channels. One output channel was connected to an analog-to-digital converter in a micro-computer. The other output channel was differentiated by an analog differential circuit. Both outputs were converted into sequences of digital signals. The differentiated signal was used to detect the beginning of each R wave. The original ECG signal was used to detect the time of the local maximum value during the period of R wave. The time interval of the two adjacent R wave peaks was calculated. The sampling frequency was 25 kHz (0.04 ms sampling interval). There were about 2.16 x 10^9^ original data points in each 24-hour ECG recording. All R-R intervals were detected in real time when the ECG signal was digitized. There were about 10^5^ R-R intervals in each 24-hour ECG recording. Only the R-R intervals were stored on a computer hard drive, with values rounded to the nearest ms, such that the effective sampling rate in RR interval was 1 kHz. Programming in assembly language for the data collection was performed by one of the coauthors (NQZ). An R-R interval was regarded as a premature beat and omitted if it deviated from the previous qualified R-R interval value by more than 30%, as with previous studies (e.g., [1]). Since premature beats were rare (<0.1%) and inconsequential in these data by virtue of our subject inclusion criterion (see above), no attempt was made to interpolate the omitted data.

1. Huikuri HV, Valkama JO, Airaksinen KE, Seppanen T, Kessler KM, et al. (1993) Frequency domain measures of heart rate variability before the onset of nonsustained and sustained ventricular tachycardia in patients with coronary artery disease. Circulation 87: 1220-1228.
